# Deciphering whipple’s disease complexity

**DOI:** 10.1007/s10238-026-02064-z

**Published:** 2026-02-06

**Authors:** Jakub Korybski, Jakub Zelig, Shreya Narayanan, Wojciech Blonski, Kalina Milena Kazimierski, Jan Hendrik Dierkes, Hanna Lidia Popiela, Augustin Patrick Gabriel, Katarzyna Neubauer

**Affiliations:** 1 Jan Mikulicz-Radecki University Hospital, Wroclaw, Poland; 2 Department of Gastroenterology, Hepatology and Internal Medicine, Jan Mikulicz-Radecki University Hospital, Wroclaw, Poland; 3 University of South Florida, Tampa, Florida USA; 4 Division of Gastroenterology, James A. Haley Veterans Affairs Hospital, Tampa, Florida USA; 5 Division of Digestive Diseases and Nutrition, Joy McCann Culverhouse Center for Swallowing Disorders, Tampa, Florida USA; 6 University Hospital Limerick HSE Midwest, Limerick, Ireland; 7 The Rotherham NHS Foundation Trust, Rotherham, UK; 8 Present Address: Department of Gastroenterology, Hepatology and Internal Medicine, Wroclaw Medical University, Wrocław, Poland

**Keywords:** Whipple’s disease, Tropheryma whipplei, Gastroenteritis, Arthralgia, Culture negative endocarditis

## Abstract

Whipple’s disease is a sporadic infectious condition, with an incidence rate of approximately 1 per million individuals. The causative agent is the gram-positive bacterium *Tropheryma whipplei*. The disease manifests with a wide range of clinical symptoms, including non-specific presentations such as diarrhea, arthralgia, and fever, as well as the more pathognomonic lipodystrophy. This diversity in presentation poses a significant diagnostic challenge even for experienced clinicians. Our review aims to provide an updated overview encompassing the latest insights into Whipple’s disease, focusing on epidemiology, pathophysiology, genetic predisposition, clinical manifestations, diagnosis, immune reconstitution inflammatory syndrome, and treatment. Herein, we have additionally explored many of the confounding factors in the diagnosis and management of Whipple’s disease, including the variable presentations among patients colonized by *Trophyrema whipplei* as well as the limitations of current treatment options, and underscore the need for further research and guidelines related to this complex disease process.

## Introduction

*Tropheryma whipplei (T. whipplei)*, the causative agent of Whipple’s disease (WD), is an insidious bacterium capable of disseminating to various tissues, causing multisystemic granulomatous disease. WD can involve multiple organs; nonetheless, the ones primarily affected are joints [1], the mucosa of the small intestine, and the central nervous system (CNS) [2]. The first description of the *T. whipplei*-induced condition dates back to 1907 when George H. Whipple, a pathologist, wrote an autopsy report describing a 36-year-old patient suffering from a group of nonspecific symptoms. Amongst the findings described by Whipple were lesions of the aortic valve, fatty depositions in the innermost tunic of the intestinal wall, permeation of foamy macrophages into mesenteric lymph nodes, and the rod-shaped bacilli embedded in the intestinal lamina propria. However, it was not until 1952 whereupon further investigation and the positive reaction to treatment with chloramphenicol that it became apparent that *T. whipplei* was, in fact, the causative agent [3].

At the beginning of the 1990 s, a characterization of this bacterium based on molecular phylogeny was achieved by employing rRNA PCR analysis, revealing a phylogenetic resemblance to *Actinomyces* species. Subsequent advancement regarding the understanding of the bacillus was made in 1997 when the first successful isolation of the bacteria was reported. Regrettably, over the following several years, this work was not reproduced, probably due to the fastidious nature of the organism. Didier Raoult was the first scientist to report stable isolation and subsequent cultivation of the bacteria in 2000 and 2001 using biopsy samples from the cardiac valve and duodenum, accordingly [4]. Nevertheless, the nonspecific symptoms, rare epidemiology, and varying clinical presentation render Whipple’s disease a persistently difficult clinical diagnosis.

## Epidemiology

With about 30 cases per year worldwide and an incidence of approximately 1 per 1.000.000, WD remains quite rare [5]. Interestingly, epidemiological studies suggest that *T. whipplei*, asymptomatically colonizes the gastrointestinal tract in 2–11% of individuals, and nearly half of the general population may be exposed to it during their lifetime [6]. The mean age of diagnosis is about 55 years, predominantly affecting Caucasian men [7]. There are variations in reported gender distribution, both historically and geographically. From the largest known study done in the United States, including 35,838,070 individuals about the epidemiology of WD, it appeared that there was no difference in prevalence based on sex; nonetheless, the disease appeared more frequently in Caucasians, non-Hispanics, and individuals older than 65 years old [8]. On the contrary, a German analysis reported a 22% increased prevalence in women compared to men [9], whereas another study indicated that it mainly affects Caucasians preferentially males in 85% of cases [10]. A retrospective, observational study from Italy revealed that the overall prevalence of *T. whipplei* in stool samples analyzed with PCR was 6.9%, with a higher rate in children [11]. A Korean study including 252 patients noted the prevalence of *T. whipplei* in duodenal biopsy tissue samples was estimated to be less than 0.4% [12, 13]. A subsequent prospective study revealed that 25% of included subjects had persistent intestinal or oral colonization with the longest duration of intestinal colonization exceeding 6 years, and 25% had intermittent colonization [14].

Most cases have been diagnosed in North America and Western Europe, which does not necessarily indicate higher prevalence amongst these demographics, though it may display the result of increased access to healthcare and advanced diagnostics. Carriage is hyper-endemic in rural Africa, which may be linked to clean water access or less effective water treatment [15]. Geographical provenance in Senegal is up to 75% seen in children from 0 to 4 years, 30% in 5–10 years of age, and 20% in 11–99 years of age [16].

Generally, transmission occurs through feces and saliva, although the bacterium has been isolated in water, which might suggest that the fecal pathway is not the sole mode of transmission [9]. Populations at risk of higher prevalence include sewer workers (26%) and individuals who may be affected by unsanitary conditions (i.e. homeless 13%). Strong evidence correlating *T. whipplei* to environmental bacteria was highlighted by the finding of *T. whipplei* DNA in 25 out of 38 (66%) sewage samples in Germany detected by PCR and hybridization. To support this evidence, a study done in residential and agricultural Austrian communities, examining influx to a sewage treatment plant, revealed 17 of 46 (37%) PCR-positive results for *T. whipplei* [17].

Transmission seemingly occurs orally and further accumulates in lymph nodes, thereafter leading to hematogenous spread to organs. Person-to-person transmission has not yet been proven. Although in a study of 26 French family members, 9/26 presented either with WD (5) or were asymptomatic carriers (4). The French case series therefore may point to intrafamilial circulation or genetic predisposition as a role in the pathogenesis of Whipple’s disease [18].

## Microbiology

*Trophyrema whipplei* originates from the Greek trophy (food) and eryma (barrier), because of the malabsorption frequently observed in the disease [3]. This gram-positive, fastidious, rod-shaped, microaerophilic [19], non-sporulating [20] bacterium, is grouped together with actinomycetes and is distinctive in its extensive genetic heterogeneity. The organism is both Period Acid Schiff (PAS) and diastase positive. *T whipplei* is unique as it is one of the slowest growing human pathogenic bacteria, taking up to 18 days to generate. Notably, and directly correlating with the spectrum of the clinical presentation, this bacterium has the capability to readily cross the blood-brain barrier. With immense adaptiveness and resistance, *T. whipplei* is most commonly found in macrophages intracellularly [21] but is also able to survive in extracellular environments [18].

There is a belief that *T. whipplei* demonstrates parasitic action, as it lacks essential biosynthetic pathways, including impairment or lack of 16 amino acids and TCA cycle genes, which are found in other bacteria. The replication of these bacteria appears to be dependent on intracellular persistence or symbiosis with other organisms [18]. Several repeated genome sequences are found during chromosomal analysis of the bacteria, which are postulated to be involved in recombination events with regions encoding surface proteins. The presence of these surface proteins is believed to play a vital role in supporting the bacteria’s ability to evade the immune system [21].

## Pathophysiology, host immunological Response, and immune reconstitution inflammatory syndrome

The immune response to *T. whipplei* infection is characterized by an interaction between immune recognition and evasion mechanisms, culminating in immune tolerance rather than effective pathogen clearance [22, 23]. This complex response, involving dendritic cells, macrophages, and T cells, features a notable impairment in Th1-mediated immunity, which is essential for battling intracellular pathogens [24].

According to Moos and Schneider’s model for pathogenesis, the primary infection probably occurs in youth through person-to-person transmission, leading to self-limiting gastroenteritis. In typical cases, after the primary infection, symptoms subside, and the immune system forms protective humoral and cellular immune responses. However, in patients developing Whipple’s disease, the humoral and mucosal T helper cell response is impaired [7].

This complex response, involving dendritic cells, macrophages, and T cells, features a notable impairment in Th1-mediated immunity, which is essential for battling intracellular pathogens [24]. DCs in patients with WD exhibit reduced maturation and impaired production of IL-12, a critical cytokine for Th1 polarization [23]. The pathogen appears to manipulate DCs, reducing the expression of maturation markers like CD83 and PD-L1, which compromises their ability to prime effective T cell responses and diminishes the activation and proliferation of Th1 cells [23, 25]. Concurrently, activated intestinal macrophages, while able to ingest the bacteria, are unable to kill them or induce a protective immune response [17]. In patients with WD, tissular macrophages exhibit an M2 alternatively activated phenotype, characterized by high CD163 and stabilin-1 expression [24]. The bacterium interferes with phagosome maturation, placing itself in the late acidic phagosome, which no longer fuses with lysosomes, thereby impairing its microbicidal functions [17]. The M2 phenotype, which includes lysosomal acid phosphatase inhibition and reduced CD11b expression, results in an impaired degradative ability [24]. Furthermore, these macrophages demonstrate reduced oxidative burst and nitrite production despite normal phagocytosis, leading to the survival and replication of *T. whipplei* within these cells [24–27].

This functional defect is reinforced by the cytokine environment. Both impaired DCs and M2 macrophages produce less IL-12 [24]. A reduced quantity of IL-12 yields a lower production of interferon-gamma (IFN-y) by NK cells and T cells, thus further impairing macrophage activation and function. The cytokine milieu is strongly biased towards a Th2 profile, characterized by elevated levels of IL-4 and IL-10, which further suppress pro-inflammatory responses and CD4 + T cell-mediated immunity [24, 26].

In this environment, *T. whipplei* uses specific mechanisms to survive. Bacterial replication in macrophages is linked to the induction of apoptosis via the extrinsic pathway (caspases 8 and 10) [17, 28]. IL-16 plays a key role, acting as a growth factor for the bacterium, an inducer of apoptosis (along with IL-1b and pro-apoptotic mediators), and a factor promoting macrophage deactivation [5, 17, 28]. IL-16 also down-regulates thioredoxin and glutaredoxin, which are critical for bacterial killing in monocytes, explaining the difference in replication capability between these cell types [27]. The result is a profound immune dysregulation, where the pathogen manipulates the host’s immune system to create an environment facilitating its survival, replication, and dissemination [24–26].

Immune reconstitution inflammatory syndrome (IRIS) is an example of an aggravated reaction caused by the reconstitution of the immune system, ultimately leading to dysregulation in the inflammatory responses. It can be observed as related to the treatment of various microbial infections, including WD, where is seen in around 10%−20% of patients [29, 30]. A patient population particularly predisposed to developing IRIS includes individuals with immunosuppression of various origins, such as those receiving glucocorticoid therapy for inflammatory joint pain which, in the early stages of WD, may mimic rheumatoid arthritis.

After the withdrawal of immunosuppressive treatment, there is a rebound of otherwise decreased CD4 + cells. The primary determinant affecting immune reconstitution inflammatory syndrome (IRIS) pathogenesis is the Th1 cell and the associated outbreak of Th1 response cytokines. The cytokine dynamics during IRIS reveal a shift from elevated IL-10 levels pre-treatment to a decline during IRIS, accompanied by the restoration of Th1 cell responses that were previously diminished due to the infection. This inflammatory response mostly manifests in inflamed tissues, especially the duodenum, where insufficient repopulation of Tregs aids tissue damage. The phase of immune restoration is represented by an abundance of naive CD45RA + T cells and CD31 + cells, contrasting with HIV-related IRIS where memory T cells are predominant. Notably, IRIS in the setting of WD appears to be navigated by mechanisms independent of pathogen-specific responses as *T. whipplei*-specific T-cell reactions remain absent. The decrease in peripheral memory CD45RO + T cells suggests their migration to extravascular tissues, perpetuating local inflammation despite successful antimicrobial therapy [22].

Yet, IRIS in the course of WD demonstrates unique features that make it distinct from IRIS resulting from other infections. The main causative factor of IRIS in WD is the immunosuppressive ability of *T. whipplei* itself, which permits the accumulation of higher loads of bacterial antigens in the various tissues [22, 29, 30].

Another consequence of IRIS is the abrupt and synergistic activation of TNF-α and IFN-γ. Certain publications describe that such synergistic cytokine surge leads to disruption of tight-junction proteins and induces epithelial apoptosis, thereby contributing to increased intestinal permeability and worsening of gastrointestinal symptoms [31]. This is intriguing, given that some antibiotics used to treat *T. whipplei* infection also can cause histological lesions to the small intestine [32], and subsequent damage to the tight junctions of the intestinal wall, inducing leakage and subsequent rise of lipopolysaccharide levels in the peripheral blood, initiating potential dysregulation in T cell reconstitution.

The clinical manifestation of IRIS in the course of WD varies based on the primary location of the pathogenic load. The most common presentations include fever and arthralgia. On top of that, IRIS can also involve lungs, skin (appearance of subcutaneous erythematous nodules), eyes (pain, pseudo-tumors located in eye sockets, diplopia, proptosis, or vision loss following hormonal treatment), joints, CNS, and lymph nodes. To make a definite diagnosis, the patient must have 2 out of 3 positive diagnostic tests confirming WD, and the presence of negative PCR while presenting with symptoms [31].

Effective management of IRIS remains problematic and predominantly relies on empirical treatments with glucocorticoids, thalidomide, and infliximab. Glucocorticoids are commonly used to control severe symptoms, although their efficacy varies and use is accompanied by potential risks, especially in cases linked to tuberculosis. Its use in the case of IRIS resulting from WD is controversial, as it has the potential to exacerbate the infection or even trigger the IRIS itself. Thalidomide, proposed as a first-line therapy, shows promise in managing IRIS-related complications like erythema nodosum reactions, while infliximab has demonstrated efficacy in specific instances. However, the current therapeutic landscape lacks robust evidence to guide treatment decisions, highlighting the critical need for well-designed clinical trials to establish optimal therapeutic approaches. Monitoring IRIS involves assessing inflammatory biomarkers such as LBP, LPS, sCD14, and various cytokines (IL-6, CCL2, CCL5, CX3CL1) to track disease progression and response to treatment. Further research is essential to refine diagnostic and monitoring strategies [31].

### Genetic predisposition/immune defects

Host susceptibility to *T. whipplei* appears to result from both specific and nonspecific immune alterations. Patients with WD present with a reduced number of CD4 T cells and specific antibodies against *T. whipplei* compared to healthy individuals [5]. In chronic carriers, some individuals appear to be protected from reinfection as they have a strong immune response, whereas those with a low immune response tend to be recolonized by another *T. whipplei* strain [16]. Lifelong relapses and reinfections in patients with WD suggest specific *T. whipplei* immunodeficiency. HLA alleles DRB*13 and DQB1*06 were more often found in patients with WD and are proposed to influence individuals acquiring chronic infection [5]. Moreover, there is a hypothesis between WD and autosomal dominant IRF4 R98W deficiency, based on a study of 4 individuals of a French kindred where all members have WD. Additionally, in the same French family, 5/26 members were asymptomatic carriers but also had the IRF4 R98W mutation. It has been suggested that the immune system’s ability to respond to an infection is mediated by the IRF4 gene. This gene has been demonstrated to play an important role in such functions [6, 33]. It is still unclear if the infection alone impairs IL-12 production or if it is a combination of the infection and genetic predisposition [21].

However, general immunosuppression of any origin, such as the chronic use of immunosuppressive drugs or Human Immunodeficiency Virus (HIV), may also predispose individuals to *T. whipplei* infection or its reactivation. This observation is justified by the fact that *T. whipplei* was found to have prevalence up to twice as high in HIV-positive patients [34], particularly in the pulmonary regions [35, 36]. Similarly, observations of human monocytes exposed to *L. pneumophila* show that autocrine *TNF* secretion enhances intracellular killing. Thus, therapeutic *TNF* inhibition may suppress phagolysosome fusion, allowing for greater intercellular bacterial replication [37].

### Clinical manifestations

**Fig. 1:**
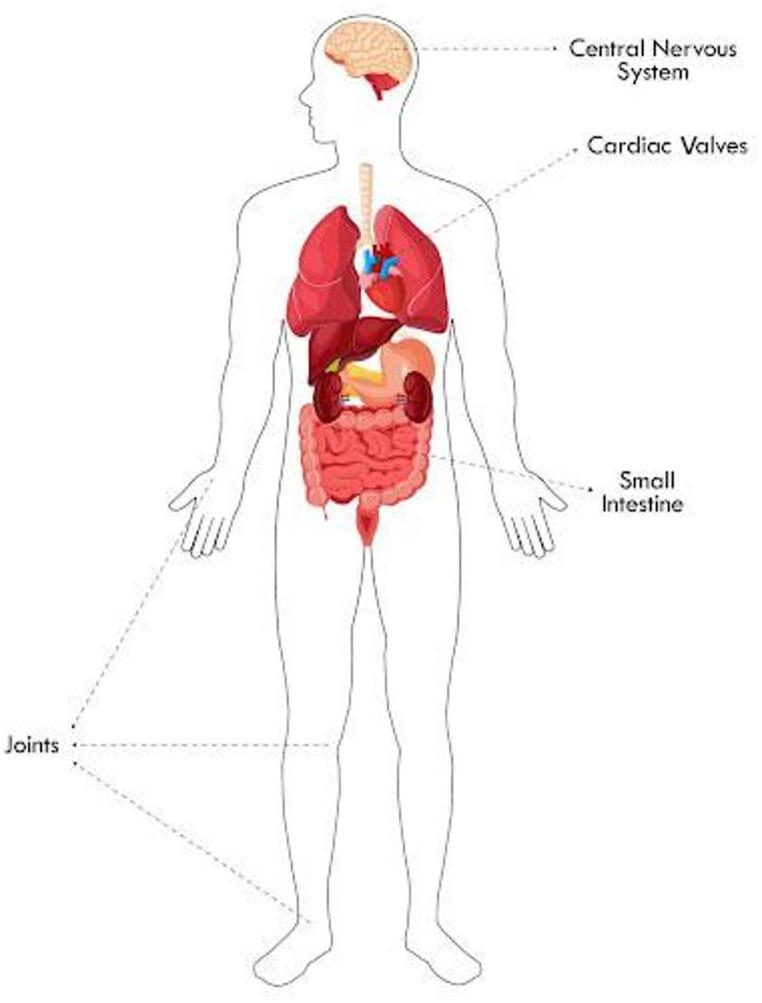
Diagram illustrating the prevalence of organ involvement

There are numerous classifications of WD, including classic WD involving the gastrointestinal tract, isolated *T. whipplei* endocarditis, and isolated neurological WD [38]. Other divisions include a gastrointestinal, extraintestinal presentation or acute phase infection (Fig. 1)[2].

### Acute infection

Typically, the infectious symptomology begins with diarrhea, abdominal pain, fever, fatigue, malaise, and less frequently nausea or vomiting [9, 18]. The acute illness, resulting in bacterial clearance and seroconversion, ultimately leads to a cured patient who becomes a chronic asymptomatic carrier [5]. A majority of patients with acute sickness present with a high bacterial load in their stool (> 104/g in stool). In a study done in 2007 involving 204 patients from two villages in rural Senegal, 13 patients were found to have *T. whipplei* bacteremia, confirmed by positive blood cultures. All 13 patients exhibited a febrile state and sleep disturbances, and 10 also presented with a concomitant cough. Seasonality was noted in this study, with all the cases occurring from June to October, with manifestations such as headache (69%) and cough (36%) [16]. In a separate study conducted in France, 241 children aged 2 to 4 years were evaluated, and *T. whipplei* was identified in 36 cases, primarily manifesting as gastroenteritis [17].

Pneumonia is a rare but classic manifestation that presents with an isolated cough. It appeared that the presentation of pneumonia caused by Whipple’s disease was most often seen in individuals with low CD4 counts, immune suppression, and aspiration pneumonia. After the point of acute infection, in predisposed individuals, the disease can take a turn and surface as classic WD or chronic localized infections.

### Classic chronic WD

Classic chronic gastrointestinal (GI) WD is the most frequently diagnosed subtype of the disease, in which the primary feature is impaired intestinal absorption. The chronic diarrhea, a hallmark symptom, typically presents as voluminous, malodorous, steatorrhea resulting from fat malabsorption due to infiltration of the small intestinal lamina propria by PAS-positive macrophages [39, 40]. This mucosal and lymphatic disruption precipitates a severe protein-losing enteropathy, with subsequent hypoalbuminemia and peripheral edema. In advanced stages, consequences are frequently multisystemic extending beyond the progressive weight loss. Deficiencies of fat-soluble vitamins, may lead to coagulopathy, osteomalacia or osteoporosis, and night blindness. Additionally, impaired absorption of electrolytes and minerals often causes hypocalcemia, hypokalemia, and iron-deficiency anemia, contributing significantly to the systemic morbidity of the disease [38, 41].

Even though WD is most often found in the GI tract, there may be an effect on other organs as well. One of the manifestations preceding gastrointestinal symptoms includes the beginning or prodromal stage that presents with arthralgia, arthritis, and fatigue. Between the onset of symptoms in the prodromal phase and the steady state stage, which is associated with a significant decrease in weight and diarrhea, there is an average of 6–8 years. The time range varies depending on whether the patient is immunocompromised, typically resulting in a shorter onset [17]. Manifestations arising in the steady state stage leading to a clearer clinical picture include gastrointestinal symptoms (diarrhea 70–85%, abdominal pain 50–90%), arthralgias (mainly bilateral and symmetrical affecting, 57% large peripheral joints, 70–90%), anemia (75–90%) and weight loss (80–90%) [2, 42]. Additional manifestations include skin pigmentation, bone involvement, lymphadenopathy, and fever. This is worth highlighting, as the manifestation of the immune reconstitution inflammatory syndrome in the course of WD encompasses an analogous clinical presentation, incorporating arthralgia and fever. Thus, this general manifestation can easily result in misdiagnosis. Therefore, in the initial weeks of therapy, the physician should be aware of the possible risk of the development of immune reconstitution inflammatory syndrome, which in many cases may mimic the classical symptoms of WD creating false suspicion of the recurrence of the disease, although when ignored, can lead to fatal consequences for the patient.

Arthralgia episodes are sudden in onset and intermittent, typically only lasting a few days at a time. Notably, these arthralgias tend to be migratory among the large joints and are non-deforming, in contrast to many of the arthritides noted with rheumatologic conditions. Continuous pain in the same joints is, conversely, less suggestive of WD. Likewise, if the inflamed joints are aspirated, the joint fluid analysis is typically non-inflammatory or only mildly inflammatory with negative cultures. Synovial biopsies may show PAS-positive macrophages. The initiation of immunosuppressive therapy for presumed inflammatory or autoimmune arthritis may precipitate the rapid development of systemic symptoms [7, 43].

The classic cardiac manifestation of WD is a culture-negative infective endocarditis. Endocarditis in WD tends to favor the aortic valve, though disease can be noted in any of the valves or in multiple valves simultaneously. Related to the difficulty in securing a diagnosis, which may lead to delays in initiation of therapy, many patients do require surgical intervention for valvular disease due to WD [44].

Central nervous system (CNS) manifestations of WD and neurological involvement are the most serious and notable chronic manifestations of classic WD, presenting in 6–63% of patients, and increasing complexity of the therapeutic course related to the need for antimicrobial therapy that penetrates the blood-brain barrier [5]. The neurologic symptoms are often various and intermingled. Commonly, these include cognitive impairment, ocular WD, myorhythmias including myoclonus or oculomasticatory myorhythmia, extrapyramidal symptoms, meningoencephalitis, or even coma [45]. 

It has also been suggested that pulmonary colonization with *T. whipplei* may trigger pulmonary marginal zone lymphoma [46]. In addition, WD can present as an isolated pulmonary disease without gastrointestinal involvement, especially in immunosuppressed patients with compromised lungs. This may manifest as pulmonary nodules, interstitial changes, patchy infiltrates, cavitation-like changes, atelectasis, pneumonia, pleural thickening or even pleural effusions on radiographic evaluation [47, 48].

### Localized endocarditis

Beyond the classic presentation of WD with cardiac involvement, isolated endocarditis is now considered a distinct entity; however, existing case reports have been limited by suboptimal characterization and reporting [7, 49]. Localized endocarditis is the most common form of localized infection, presenting without histologic digestive lesions. Generally, it is preceded by arthritis or arthralgia. Diagnostics for isolated infective endocarditis due to WD remain quite a challenge, as definitive diagnosis in the absence of other serum or histologic positivity would be through cardiac valve sampling at the time of surgery [17].

### Localized central nervous system Whipple disease

CNS is involved in 90% of cases of WD, but neurological manifestations are evident in only 10–43% [5, 50], and are mainly represented by cognitive impairment, psychiatric dysfunction, sleep disturbances, oculo-masticatory myorhythmia, oculo-facio-skeletal myorhythmia, seizures, and ataxia, while medullary manifestations are rare, and few data are present in the literature [51, 52]. Though patients may present with varying symptoms, including cognitive and psychiatric changes, the pathognomonic neurologic changes include oculofacial-skeletal myorhythmia or oculomasticatory myorhythmia. A highly specific CNS WD triad comprises dementia, supranuclear ophthalmoplegia, and myoclonus [5]. In WD, encephalitis typically manifests with heterogeneous involvement of the central nervous system, occurring in both isolated and classic forms of the disease [15]. Asymptomatic involvement of the CNS is detectable by PCR for *T. whipplei* in the CSF in 39–50% of all WD cases, even without clinical signs of neurological involvement [7, 53, 54].

### Prognosis

The prognosis of WD is generally favorable if diagnosed early and treated appropriately with prolonged antibiotic therapy (~ 8.5% mortality) [55]. Untreated WD is almost universally fatal due to progressive multi-organ involvement, particularly gastrointestinal, cardiac, and neurological complications [56]. Even with treatment, WD can be complicated by infectious, neoplastic and thrombolytic disorders. Moreover, relapses can occur, especially in patients with CNS involvement, immunosuppression, or incomplete therapy. Early initiation of antibiotics significantly reduces the risk of mortality and long-term complications. Long-term follow-up with periodic clinical assessment and, if indicated, PCR testing of blood or tissue samples is recommended to monitor for recurrence [55].

Overall, early recognition and sustained therapy are key determinants of a good outcome, while delayed diagnosis or CNS involvement worsens prognosis.

### Pathology

Although Whipple’s disease can present as a multi-organ disease, classic WD is found in the small intestine, especially the duodenum, where several characteristic features emerge. On macroscopic examination, the mucosa often appears unremarkable, but deviations from normalcy sometimes become evident. The mucosa may take on a pale yellow color and become dilated, thickened, and rigid. Additionally, a fibrinous exudate may be observed on the peritoneal surface. Notably, the villus tips exhibit a whitened appearance. Furthermore, mesenteric and para-aortic lymph nodes show enlargement. These findings may coincide with duodenitis of the duodenal mucosa. In some instances, the presence of reddened crinkle tips, swollen villi, and aphthous ulcerations provides a subtle hint of gastrointestinal involvement [57].

Histologically, Whipple’s disease is characterized by the presence of distinctive macrophages harboring roundish inclusions, formerly referred to as sickle particle-containing (SPC) cells. These macrophages proliferate in a characteristic zigzag pattern and exhibit a unique staining property: they light up red in the Periodic Acid-Schiff (PAS) stain. Identifying these PAS-positive cells is considered typical for Whipple’s disease. Infections such as *Mycobacterium avium* complex and *Rhodococcus*, which also stain PAS positive, may confound or delay the diagnosis. Other conditions that stain positive for PAS include Gaucher disease and crushed Brunner glands of the duodenum. Notably, these cells are not confined to the CNS or gastrointestinal tract but are found distributed throughout various affected organs, underlining the systemic nature of the disease [58].

Microscopic analysis of small bowel tissue demonstrates villus distension due to infiltration by foamy macrophages. These macrophages replace the lamina propria and may extend into deeper layers such as the muscularis mucosae or submucosa, contributing to structural alterations and functional impairment of the intestinal tract [59]. Furthermore, microscopic examination unveils secondary manifestations of Whipple’s disease, such as lipodystrophy. Within affected tissues, rounded empty spaces are observed, containing neutral fat [60].

In the CNS, lesions display necrosis accompanied by an inflammatory infiltrate predominantly composed of T cells (CD3 and CD8 positive) and B cells (CD4 and CD20 positive). Additionally, astrogliosis, indicative of reactive changes in astrocytes, is observed, reflecting the brain’s response to the underlying pathology.

### Diagnosis

In the early 2000 s, *T. whipplei* was successfully cultured in human fibroblast cells, allowing for genome sequencing and the development of immunohistochemical staining and diagnostic PCR assays [61]. Currently, numerous methods are used to detect the bacterium, including quantitative PCR, histopathology, immunohistochemistry, and fluorescence in situ hybridization (FISH) on biopsy samples [21]. A positive diagnosis generally requires a combination of tests, often two positive results from key methods.

A primary component of the diagnostic criteria is PAS staining, which detects foamy macrophages in biopsy specimens of involved tissues [17, 21]. However, PAS staining also detects Mycobacterium avium intracellulare, Rhodococcus equi, Bacillus cereus, Corynebacterium, Histoplasma, and fungi, rendering it non-specific for *T. whipplei* [17].

Consequently, definitive diagnostic criteria should include immunohistochemical staining with *T. whipplei* antibodies obtained from intestinal samples or other clinically relevant systems [5]. This diagnostic method has proved to be more sensitive and specific than PAS, potentially leading to a more accurate finding of *T. whipplei*. Alternatively, molecular confirmation is key.

**Fig. 2:**
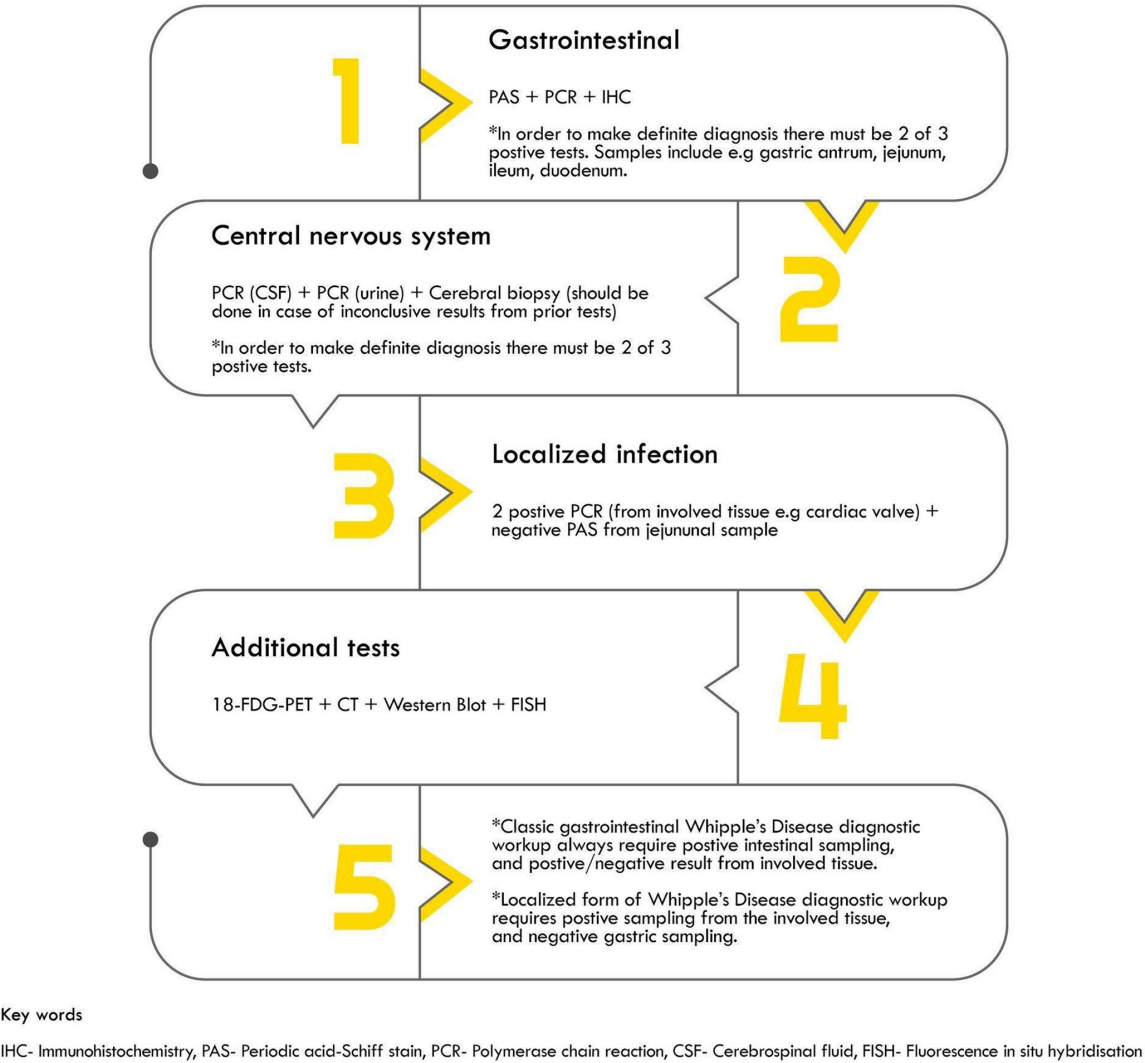
Flow diagram of diagnostic pathway for diagnosis of Whipple’s Disease

A generalized diagnostic pathway, particularly for localized infections, involves negative PAS staining in a small bowel biopsy coupled with two positive PCR results from the affected system [21] (Fig. 2). An additional limitation of sampling is the unequal bacterial distribution within the small intestine; consequently, additional biopsies from other sites, such as the gastric antrum, jejunum, and ileum should be acquired regardless of the initial result [2].

### Small intestine

In patients presenting with the classic triad of weight loss, chronic diarrhea, and arthralgia, histopathological analysis of small intestinal mucosal biopsy specimens remains the cornerstone of diagnosis. The preferred tissue sample should be acquired from the duodenum or proximal jejunum. Positive samples demonstrate distended lamina propria macrophages filled with PAS-positive, diastase-resistant granules [62, 63]. These foamy macrophages often compress villi, producing characteristic blunting and broadening of the mucosa [63].

However, PAS staining alone is not pathognomonic. Therefore, confirmatory testing with Tropheryma whipplei–specific PCR or IHC is essential for diagnostic validation [2, 21]. PCR performed on small-intestinal biopsy tissue achieves sensitivities exceeding 90% in classic WD and is regarded as the molecular gold standard [64]. Immunohistochemical staining using polyclonal anti–T. whipplei antibodies provide both morphologic and pathogen-specific confirmation, detecting bacilli within macrophages and extracellularly [62]. Electron microscopy, though now rarely performed, can further demonstrate the characteristic trilaminar cell wall of *T. whipplei* bacilli, supporting the diagnosis in equivocal cases [57].

A positive result from stool or saliva PCR alone must be interpreted cautiously, as asymptomatic carriage of *T. whipplei* occurs in up to 4% of healthy individuals [12, 65]. Because bacterial DNA can also be transiently detected in the stool or saliva, only a positive PCR from intestinal tissue in conjunction with typical histopathology remains the definitive diagnostic criterion for classic WD [15].

### Cardiac valves

Cardiac manifestations of WD are most frequently due to *T. whipplei* endocarditis, which can occur with or without concurrent intestinal disease and often presents as blood culture–negative infective endocarditis [11, 12]. The diagnosis is challenging because serological and culture-based methods are consistently negative, and echocardiographic findings are nonspecific. PCR amplification of *T. whipplei* DNA from excised valvular tissue or blood samples has become the gold standard for etiologic confirmation [3]. In most cases, PCR of blood is negative, reflecting a low circulating bacterial load, whereas positive PCR results from resected valves or embolic fragments provide definitive evidence of infection [66]. Histopathological examination reveals macrophage infiltration of the valvular tissue containing PAS-positive, diastase-resistant granules, although such findings may come late in the course of the infection, oftentimes requiring open-heart surgery [67]. Immunohistochemistry and electron microscopy further support diagnosis by visualizing bacillary forms of *T. whipplei* within macrophages or extracellularly [68]. The combination of clinical suspicion, PAS-positive valvular histology, and confirmatory PCR from sterile cardiac material remains the diagnostic basis. Because isolated *T. whipplei* endocarditis can occur in the absence of gastrointestinal symptoms, molecular testing should be systematically included in the diagnostic algorithm for all cases of culture-negative endocarditis [66, 67].

### Cerebrospinal fluid and cerebral biopsy

Neurological WD represents one of the most diagnostically challenging forms, as it may occur in isolation or alongside systemic infection. CNS involvement can manifest years after initial systemic symptoms, with presentations ranging from cognitive decline and ophthalmoplegia to seizures or psychiatric disturbances [16, 17]. Histopathologic evaluation of brain biopsy material remains the diagnostic gold standard, showing perivascular macrophages containing PAS–positive, diastase-resistant granules, often accompanied by gliosis and neuronal loss [69]. However, brain biopsy is rarely achievable; therefore, molecular diagnostics have become crucial.

PCR detection of *Tropheryma whipplei* DNA in CSF offers a sensitive and specific, though not infallible, diagnostic method. In addition to PAS staining, specific 16 S rRNA PCR on cerebral or CSF material is also utilized [21]. Furthermore, the CSF findings in CNS WD are heterogeneous and non-specific. Some patients may have completely normal CSF, while others exhibit mild lymphocytic pleocytosis and elevated protein. Reports also describe predominance of mononuclear cells or, less commonly, neutrophils. Glucose levels in CSF are typically normal, although transient hypoglycorrhachia may occur in the setting of co-existing meningitis or complications. Because of this variability, PCR testing for *T. whipplei* in CSF should be considered even when routine CSF indications appear normal. The specificity of PCR is high, but sensitivity in CSF is variable (~ 41–63% in patients without the CNS symptoms, and if neurologic symptoms are present, it rises up to 59.1%−92%), so a negative result does not entirely exclude CNS involvement in the appropriate clinical context [39, 70, 71].

Another tool that can be utilized is the magnetic resonance imaging (MRI) of the brain, described findings include T2/FLAIR hyperintense lesions (especially in the deep grey matter, hypothalamus, medial temporal lobes), inflammatory-granulomatous foci, involvement along white-matter tracts, and mild contrast enhancement; however, MRI may also be normal, so imaging must be interpreted in the context of clinical and microbiological data [72].

In classic Whipple’s disease, the high bacterial load typically allows for effective diagnosis using PCR on fresh tissue, vitreous fluid, and cardiac valves [17]. However, in cases where symptoms are varied, and the bacterial load is lower, PCR sensitivity can be compromised [15]. Similarly, PCR testing may also appear negative after the use of antibiotics, due to a decreased bacterial load [73]. Encephalitis is challenging to diagnose, as CSF testing often yields negative PCR results. However, recent research suggests that PCR testing on urine samples may offer a non-invasive diagnostic approach for Whipple’s disease, including potential neurological manifestations [15].

When clinical suspicion is high but CSF PCR is negative, repeated lumbar puncture or tissue sampling is advised, given the patchy distribution of the pathogen [69]. Immunohistochemistry and electron microscopy, when available, confirm diagnosis by visualizing *T. whipplei* loaded macrophages in CNS tissue [21].

### Synovial fluid

When chronic, seronegative oligo- or polyarthritis presents as the sole or predominant clinical feature, the diagnostic focus must shift to PCR analysis of synovial fluid or synovial membrane biopsies. This approach is necessary because routine autoimmune serologies (such as rheumatoid factor and anti-CCP antibodies) are typically negative, and imaging reveals only non-erosive synovitis or tenosynovitis. The utility of a traditional duodenal biopsy, which looks for PAS-positive foamy macrophages, is limited in joint-limited disease; its sensitivity is low (12%) as intestinal involvement may be absent. This limitation has elevated PCR testing of *T. whipplei* DNA in synovial fluid or tissue to the key diagnostic tool, which is positive in approximately 85% of patients with localized articular WD. Synovial fluid in articular WD is frequently sterile with positive PCR for *T. whipplei*, but detailed descriptions of cell-type distribution, glucose and protein levels are sparse in the literature [65].

When feasible, a synovial biopsy for histopathology and molecular confirmation is ideal, as it provides direct evidence of bacterial presence within macrophages. In cases where duodenal biopsy and synovial PCR yield discordant results, current consensus recommends accepting two independent positive tests as sufficient for diagnosis [38, 63].

Early microbiologic confirmation is crucial, as immunosuppressive therapy administered for presumed autoimmune arthritis can accelerate dissemination and worsen prognosis. Therefore, in seronegative, treatment-refractory, or migratory arthritis, especially involving large joints, *T. whipplei* PCR of synovial fluid or tissue should be integrated into the diagnostic algorithm before escalation of immunosuppressive therapy [38].

### Vitreous aspiration

Ocular involvement in WD represents a rare but diagnostically important form that may occur in both systemic and localized CNS infections. Clinical manifestations are heterogeneous and include uveitis, retinitis, vitritis, optic neuritis, and keratitis, often with relapsing or steroid-responsive courses that may precede systemic features by months or years [74]. Because these presentations frequently mimic autoimmune or idiopathic uveitis, definitive diagnosis depends on the detection of *T. whipplei* in ocular fluids or tissue.

Conventional histopathology of ocular tissue may reveal PAS-positive foamy macrophages, but this is rarely available and lacks sensitivity in localized disease. PCR for *T. whipplei* DNA in aqueous or vitreous humor has thus become the cornerstone of diagnosis, offering high specificity and good sensitivity, even in the absence of gastrointestinal or joint involvement. In a review of culture-confirmed and PCR-verified ocular WD, PCR of intraocular fluid was positive in nearly all tested cases, whereas duodenal biopsy was often negative [74, 75].

Importantly, ocular WD may represent an isolated or relapse form after incomplete systemic treatment, and thus PCR testing of ocular samples should be performed in chronic uveitis unresponsive to immunosuppressive therapy, particularly when associated with neurologic or systemic symptoms. When available, immunohistochemistry directed against *T. whipplei* antigens can complement PCR and confirm bacterial localization within macrophages. Given the potentially blinding course and diagnostic delay ranging from 2 to 28 months, early molecular testing of intraocular fluids is strongly recommended to establish the diagnosis and guide prolonged antibiotic therapy [74–78].

### Stool carriage

The differentiation between asymptomatic *T. whipplei* carriage and active WD is a major diagnostic challenge, particularly in the PCR era. *T. whipplei* DNA can be detected in the stool and saliva of about 4% of healthy individuals [65] and up to 26% in a subgroup of sewage workers, reflecting transient colonization rather than systemic infection [3]. Thus, a single positive PCR result from non-sterile specimens cannot establish a diagnosis of WD. Quantitative PCR studies demonstrate that bacterial load is markedly higher in patients with active disease compared to asymptomatic carriers, and positivity in normally sterile sites is far more predictive of true infection [38, 64, 79]. Histopathological evidence of PAS-positive foamy macrophages in small-bowel mucosa, together with immunohistochemistry or electron microscopy confirming *T. whipplei*, remains essential for confirming systemic involvement. Current consensus recommends that WD should be diagnosed either by the combination of typical histopathology and *T. whipplei* detection by tissue PCR or immunohistochemistry, or by demonstration of the organism in at least two independent sterile sites in patients lacking classic intestinal lesions [5]. When isolated PCR positivity is found in stool or saliva without clinical signs, patients should be classified as carriers, and antimicrobial treatment is not indicated, although clinical monitoring should be present [38, 64, 79].

### Supplementary diagnostic methods

Finally, supplementary serological Western blot test, though not routinely used, can differentiate asymptomatic carriers from WD patients by using protein deglycosylated 110 kDa and assessment of lower immunological response in WD patients comparing to carriers (this due to impaired function of macrophages, and *T. whipplei* glycosylation) [7, 15]. Furthermore, 18-FDG-PET has been suggested for initial assessment and subsequent follow-up on the progression of the disease. It can identify both duodenal and cerebral involvement by revealing areas of hypometabolism and enabling comparisons between scans over time [15, 80]. Additionally, WD may also manifest with low-density lymphadenopathy on CT [81, 82].

### Associated oncological considerations

Potential contribution of microbial infections to carcinogenesis is well documented, as exemplified by *Helicobacter pylori* [83, 84] in gastric cancer and *Epstein-Barr virus* [85, 86] in lymphoproliferative disorders. Although a direct causal relationship has not been conclusively established in the case of *T. whipplei*, some data suggest its potential involvement in lymphoproliferative oncogenesis, for instance, the pulmonary subtype of marginal zone lymphoma (MZL).

Asymptomatic pulmonary colonization with *T. whipplei* has been reported in several studies. The identified microbial presence in healthy, HIV-negative individuals varies significantly across studies, typically ranging from 5% to 26% of subjects. Notably, one study reported that out of 5% initial positives, 19% showed repeat positive results in subsequent testing, indicating persistent carriage. The highest prevalence was documented in HIV-infected patients, among whom *T. whipplei* was detected in 43.4%−53.7% of specimens [87–90] (Table 1).

Pulmonary MZL, arises primarily in the lung and belongs to extranodal MZLs [91].A hallmark feature of MZLs lymphomagenesis is its association with chronic antigenic stimulation [92]. Given that *T. whipplei* induces a chronic systemic infection and has been shown to colonize the lungs, its sustained presence in pulmonary lymphoid tissue could provide the antigenic stimulus required for lymphoproliferation [46, 84, 87–90].

The NF-κB pathway regulates B-cell proliferation, differentiation, and apoptosis. In most PMZL cases (~ 60%), constitutive NF-κB activation results from somatic mutations or translocations in regulatory genes such as *TNFAIP3*, *CARD11*, or *MALT1*. In contrast, PMZL linked to chronic *Tropheryma whipplei* infection lacks such mutations, suggesting an extrinsic, bacterium-driven mechanism. Persistent antigenic stimulation by *T. whipplei* chronically activates NF-κB through BCR and pattern-recognition signaling, mimicking the effect of genetic lesions and eliminating the need for intrinsic mutations [46, 93–95].

The overlap of the epidemiological data on *T. whipplei* colonization and a molecular understanding of NF-κB pathway dysregulation suggests that *T. whipplei* may act as an infectious trigger in pulmonary marginal zone lymphoma pathogenesis. Although definitive evidence of causality remains to be established, these findings highlight the urgent need for further research to explore the therapeutic implications of targeting *T. whipplei* in the management of PMZL, potentially offering novel strategies akin to *H. pylori* eradication in gastric MALT lymphoma.

**Table 1:**
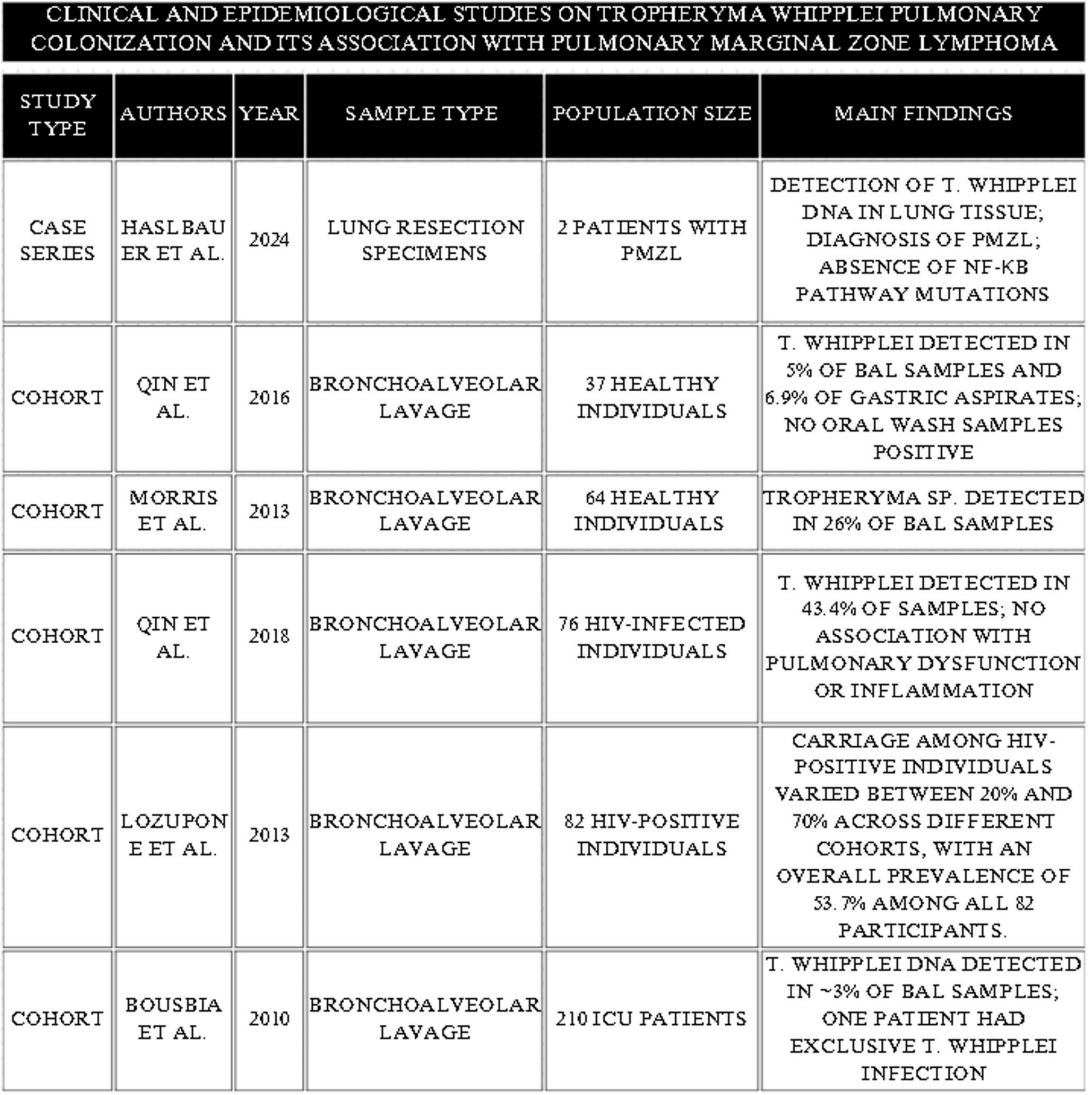
Overview of studies on *Tropheryma whipplei* colonization and association with pulmonary marginal zone lymphoma

## Treatment

Several reviews have been published regarding the management of *T. whipplei* infection, but no consensus has been reached. The currently available microbial chemotherapy recommended for the treatment of WD consists of combinations of trimethoprim/sulfamethoxazole (TMP-SMX), doxycycline, hydroxychloroquine [15], and ceftriaxone [70]. However, some reports state that Interferon-gamma can also be used due to its role in controlling intracellular types of infections such as WD, particularly in the cases of relapsing neurological disease [3, 56]. The appropriate chemotherapy and the length of treatment should be chosen based on in-vitro susceptibility of the bacteria, and the form of the disease [15]. The initial weeks of therapy are crucial. The physician should be able to recognize the potential risk of immune reconstitution inflammatory syndrome, which often imitates the typical manifestation of WD, creating incorrect suspicion of the disease recurrence. Nevertheless, the estimated risk is small and concerns mainly male patients over 65 years of age. In previous years, the majority of the literature suggested the use of TMP-SMX but this former gold standard has proven to be ineffective against *T. whipplei*. The emergence of resistance to TMP-SMX is based on two separate mechanisms: chromosomal and plasmid-mediated. Chromosomal resistance to TMP emerges from the changes in the dihydrofolate reductase DHFR enzyme, mainly stimulated by transposon Tn7 integration, that ultimately leads to thymine dependence and transformations that elevate the inhibitory constant for TMP, resulting in enzymatic overproduction. On the contrary, plasmid-mediated resistance concerns different DHFR variants that are distributed through horizontal gene transfer and cassette-mediated mechanisms, these include the dhfrI gene within the Tn7 transposon and integrons. Likewise, sulfonamide resistance at the chromosomal level generally results from modifications in dihydropteroate synthase DHPS, a fundamental enzyme in folate biosynthesis. On the other hand, plasmid-borne sulfonamide resistance is conferred by sul I and sul II genes encoding resistant DHPS variants. These genes are usually found on transposons or small plasmids, contributing to their overall dispersal despite decreased sulfonamide usage. The analyses conducted on the *T. whipplei* genome established that the bacteria voids the coding sequence for dihydrofolate reductase, which is the main enzyme target of trimethoprim, and lack of this enzyme will eventually lead to the development of resistance and ultimate failure of therapy. Both in-vitro studies and subsequent clinical outcomes confirmed failures of TMP-SMX therapies [61]. The observed failure rate varied from 9.1% up to 23%, increasing even up to 40–42% in patients with CNS involvement [96–98]. The sole indication for the use of TMP-SMX is when it is used in addition to the classical regimen in cases that present with CNS involvement [17]. Additionally, together with the natural resistance towards the antibiotic class of fluoroquinolones [99], these remain the sole occurrences of antibiotic resistance observed in *T. whipplei.* According to a study published in *The Lancet*, oral-only treatment of WD was found to be safe and non-inferior to the standard sequential intravenous-oral approach. This approach may simplify the long term patient management and reduce hospital acquired complications and costs, however due to limitations of the study, further research is required before these findings can be widely implemented in clinical practice [100, 101]. Upon consideration, of the most recent publications in the field, the classical progression of WD should be treated with doxycycline (200 mg per day PO) combined with hydroxychloroquine (600 mg per day PO) over the length of 1 year, followed by lifelong monotherapy with doxycycline (200 mg per day PO). While controversial, this prophylactic strategy is based on several factors: the potential for reinfection and relapse (less than 10% risk of relapse in patients receiving doxycycline for minimum of 12 to18 months), the low immunological response against the pathogen observed in most cases, and the proven lifelong susceptibility of patients to *T. whipplei*. Furthermore, the significant risk of relapse following treatment cessation reinforces the need for continuous strategy for patients with localized endocarditis, neurological WD, and immunosuppressed patients [15, 98, 100, 102, 103]. Although not routine, a similar strategy is used for other chronic bacterial infections, such as *Coxiella burnetii*, *Brucella* spp. endocarditis, and *Bartonella* spp. prosthetic valve or vascular graft infections [104–108]. This approach represents a rational and effective strategy to prevent recurrence and ensure long-term disease control.

In the case of a localized form of the disease (e.g.endocarditis), the suggested treatment consists of doxycycline (200 mg per day PO), and hydroxychloroquine (600 mg per day PO) for a minimum of 12 months up to 18 months. Additionally, patients should have lifetime follow-up visits [Table 2].

**Table 2:**
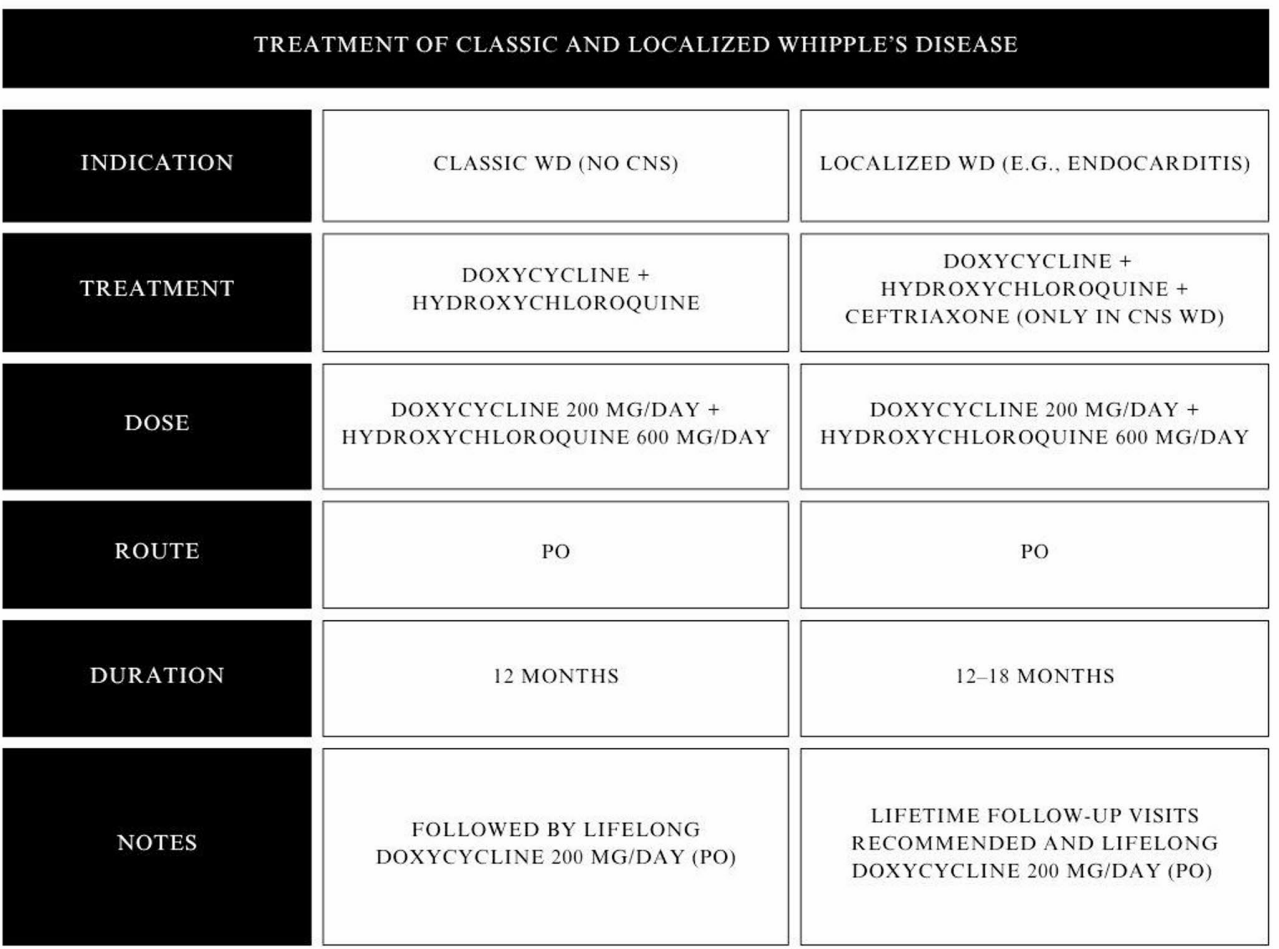
Table indicating the treatment regimens for classic and localized whipple’s disease

In the situation of WD involving CNS, the choice of antibiotics differs from the aforementioned. In this instance, intravenous ceftriaxone dosed 4 g per day for 6 weeks is recommended due to its demonstrated ability to penetrate the blood-brain barrier, followed by TMP-SMX (1600/320 mg per day PO) taken for a year [109]. Alternatively, in case of failure of the above-mentioned regimen, varied loading regimens may be considered such as cefixime 400 mg daily PO for 1–2 years, penicillin-G 1,2 million IU twice a day with intravenous streptomycin 1 g twice daily for 14 days, intravenous ceftriaxone 2 g daily with intravenous ampicillin 2 g three times per day for 14 days, or intravenous ceftriaxone 2 g twice daily with intravenous streptomycin 1 g twice daily for 14 days [3, Table 3]. However, these options require further research to validate and should be used only as a last-resort treatment in case of failures of standard therapies Table 3.

**Table 3:**
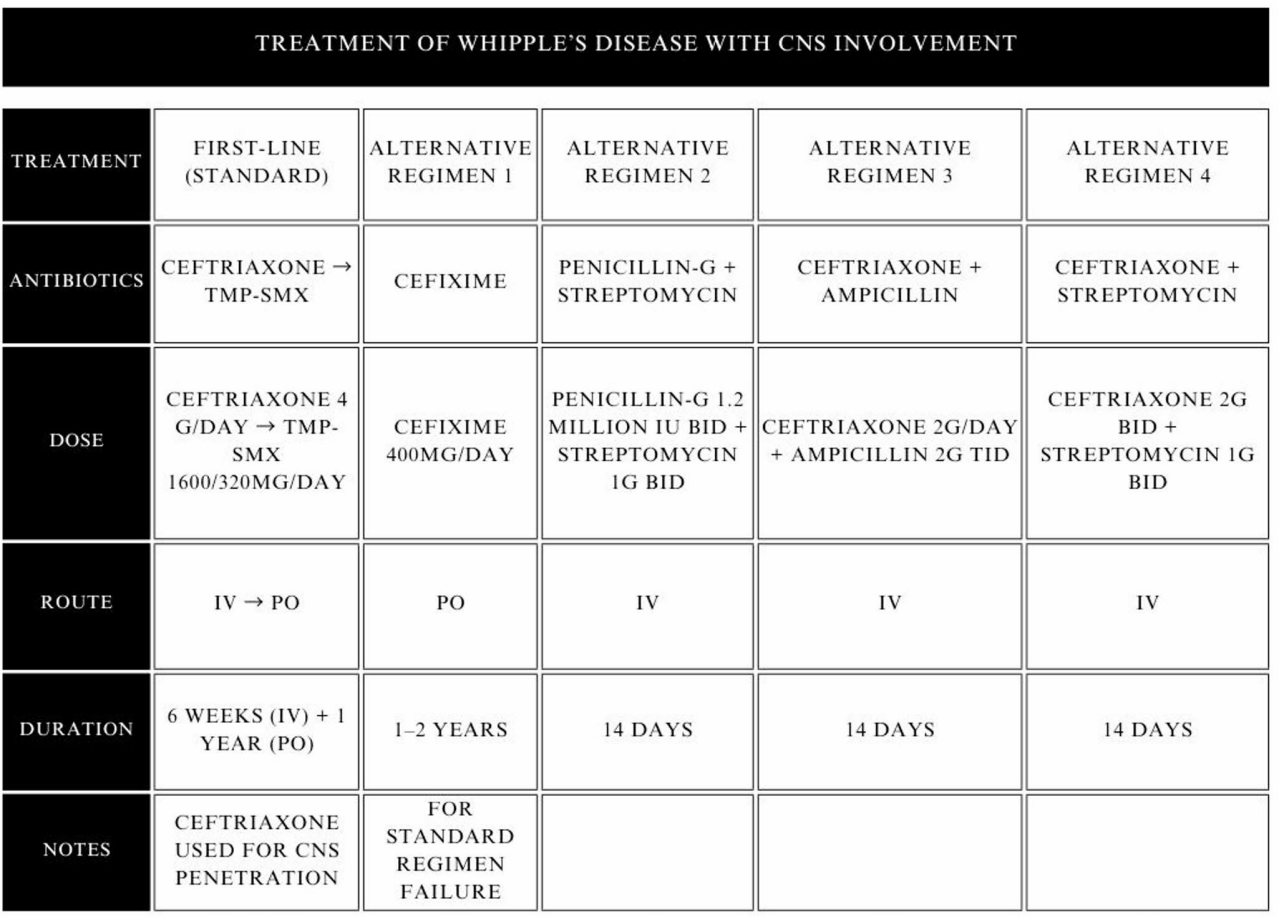
Table indicating the first-line and alternative treatment options for whipple’s disease with central nervous system involvement

The safety of therapy is a fundamental factor in drug selection, including the side effect profile and risk of complications. Common side effects and *Clostridium difficile infection* (CDI) risk are presented [Table 4].

**Table 4:**
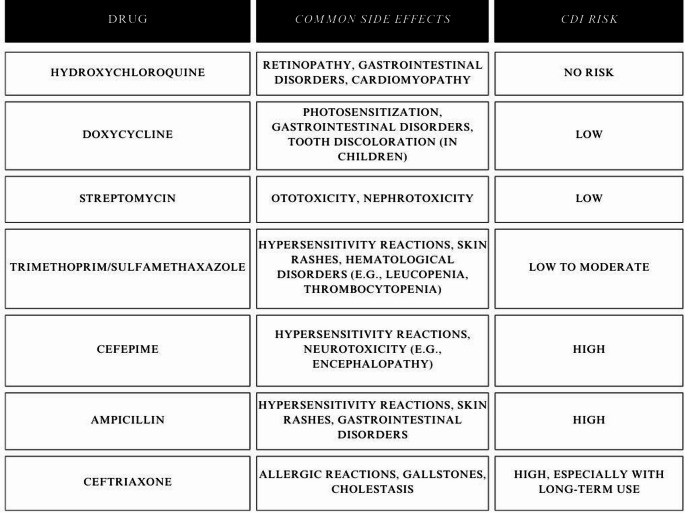
Table indicating the most common side effects of the drugs described and their relative risk of developing CDI [110]

### Microbiota alterations after WD treatment regimens

Co-trimoxazole (TMP-SMX) is a broad-spectrum antibiotic active against both gram-positive and negative aerobic bacteria. Both anaerobic and atypical bacteria tend to show resistance to this agent, with numerous reports describing strong suppression of intestinal gram-negative aerobic flora, with most of the anaerobic flora not affected by the long-term use of TMP-SMX. Conversely, there were moderate increases in the concentrations of *Candida* species and ESBL-producing strains of *E.coli* [111] Table 4.

Doxycycline expresses poor activity towards gram-positive bacteria, and moderate activity against gram-negative as well as anaerobic bacteria. It is a suitable choice for atypical bacteria and intracellular pathogens such as *T. whipplei* [110]. Researchers suggest that long-term use does not induce major shifts in the intestinal flora, pathogenic overgrowth or increase in resistant species [112, 113]. On the contrary, dissenting publications report an increase in colonies of *enterococci*, and *E. coli*, visible just after 16 weeks of treatment [114]. Moreover, doxycycline is often co-administered with a commonly used antimalarial drug, hydroxychloroquine. This drug primarily targets monocytes and lymphocytes T inhibiting their proliferation [115]. Animal studies regarding hydroxychloroquine’s effect on intestinal flora indicated that even the short-term administration of the drug leads to significant alterations in the enteric microbiome, causing changes in the bacterial compositions reducing bacterial diversity. Presently, this lacks confirmation in human-based studies and remains a hypothesis until substantiated [116, 117].

The drug used least often against *T. whipplei*, ceftriaxone, is a member of the third generation of cephalosporins and is used mainly for the infection confined to the CNS. This β-lactam demonstrates moderate bactericidal activity against gram-positive aerobes, coupled with good efficacy towards aerobic gram-negative bacteria, and some anaerobic species [110]. However, it is crucial to underline that enterococci exhibit intrinsicity towards ceftriaxone, which poses a considerable challenge in clinical practice [118]. Its usage is associated with a significant decrease in the numbers of *E. coli* and other anaerobic bacteria present in the gut, and increased risk of *Clodtridioides difficile* infection [119]. Alongside this, there was a disappearance of *staphylococci*, *streptococci*, *veillonella*, *peptococcus*, *fusobacterium*, *bacteroides*, and *bifidobacterium* [120]. Furthermore, there was an evident tendency in the direction of overgrowth of *enterococci*, yeasts, and antibiotic-resistant species [32, 120, 121]. To build upon that, the same study identified bacterial species associated with different duration of ceftriaxone use [32]. This is significant as other research confirmed that these conditions aided the proliferation of *enterococci* in the intestinal lumen before their dissemination to multiple organs such as the spleen, liver, and mesenteric lymph nodes. This is due to dysbiosis and the disruption of the integrity of the intestinal barrier following ceftriaxone usage [122].

## Conclusion

Whipple’s disease is a relatively rare chronic infectious disorder, characterized by a slow progression of symptoms that create a clinical picture lacking any pathognomonic signs. Taking into account that the symptomatology of WD widely varies and can involve one or multiple organs, *T. whipplei* infection should be excluded in cases of unexplained weight loss, chronic diarrhea, arthritis or endocarditis, especially when there is no other obvious cause. The diagnostic process requires not only histopathology and immunohistochemical staining, but tissue/fluid PCR confirmation as well. Once diagnosis is established, it is crucial to thoroughly analyze the possibility of other organ involvement, especially the central nervous system, which can be unaffected at the time of diagnosis. There are a variety of antibacterial agents effective against *T. whipplei*. Thus, the choice of treatment scheme requires analysis of tissue penetration - including the blood-brain barrier - and potential adverse effects of the applied therapeutics.

In summary, it is most important to consider Whipple’s disease in differential diagnosis and to remember that it can cause a wide spectrum of symptoms.

## Data Availability

No datasets were generated or analysed during the current study.
